# “Working on Wellness:” protocol for a worksite health promotion capacity-building program for employers

**DOI:** 10.1186/s12889-019-6405-1

**Published:** 2019-01-25

**Authors:** Mari Ryan, Lisa Erck, Leslee McGovern, Kathleen McCabe, Kevin Myers, Suzanne Nobrega, Wenjun Li, Wen-Chieh Lin, Laura Punnett

**Affiliations:** 1 AdvancingWellness, Watertown, MA USA; 2 Health Resources in Action, Boston, MA USA; 3 University of Massachusetts Lowell, Lowell, MA USA; 4 University of Massachusetts Medical School, Worcester, MA USA; 5 Kansas Health Institute, Topeka, KS USA

**Keywords:** Small employers, Worksite health promotion, Worksite wellness, Intervention planning

## Abstract

**Background:**

In the United States, worksite wellness programs are more often offered by larger employers. The Massachusetts Working on Wellness (WoW) program is an innovative, statewide capacity-building model designed to increase the number of smaller employers (200 or fewer workers) adopting health promotion initiatives. This article describes the WoW program design and approaches to recruitment, implementation, and evaluation.

**Methods/design:**

WoW provides employer training, technical assistance and seed funding, utilizing a Wellness Program Development framework based on recognized good practices. For-profit employers with 200 employees or fewer are eligible for and encouraged to apply for a Massachusetts Small Business Wellness Tax Credit. During the phase described in this paper, employer organizations applied to the program and committed to designating a champion responsible for program implementation. Interventions were to include policy and environmental supports, as well as those targeting individual behavior change through raising awareness and education. Supports provided to employers included seed grants for qualifying activities (up to $10,000 with matching required), community linkages, data collection and organization-specific feedback tools, an on-line curriculum supplemented with technical assistance, and an expert webinar series. Data collection at multiple time points, from the initial application through program completion, provides information for evaluation of recruitment, planned and completed activities.

**Discussion:**

This model is grounded in literature on good practices as well as in local knowledge about Massachusetts employers. It does not directly address the influence of working conditions, which can affect both worker participation and health behaviors. Implementation may be less successful with some organizations, such as those with many workers who are part-time or geographically distributed rather than in a centralized physical location. Program evaluation will assess the extent to which WoW achieves its goals. The data are expected to increase understanding of the needs of smaller employers and industries not traditionally implementing employee wellness programs.

**Electronic supplementary material:**

The online version of this article (10.1186/s12889-019-6405-1) contains supplementary material, which is available to authorized users.

## Background

Among American working adults, chronic diseases such as obesity, diabetes, and cardiovascular disease are highly prevalent health issues. These health problems are associated with a number of behaviors including inadequate consumption of healthy foods [1], use of tobacco products [2], and inadequate physical activity [3].

The workplace can provide an environment of social support with opportunities for direct communication with employees to support and encourage healthy lifestyle choices. American workers spend an average of 8.8 h per day in work or work-related activities [4], and many employers are now educating employees on how to adopt healthy lifestyles [5].

However, the workplaces and structures of many jobs have inherent health-diminishing aspects [6–8]. Factors such as psychosocial job strain, shift work, and excessive physical work load are related to worker behaviors such as smoking and lack of leisure-time exercise [9–12]. Thus the workplace environment itself should be designed to support these choices and to mitigate the stressors that counteract healthy behaviors [13]. Educational efforts alone are insufficient if the workplace structure, policies, and practices remain unhealthy and unsupportive of healthy behaviors.

Some employers recognize their role in providing workplace policies and practices that protect employees and advance safety, health and wellbeing [14] and are providing worksite health promotion (WHP) programs which include that goal [15, 16]. Good practices in comprehensive workplace wellness programs include health education, supportive physical and social environments, integration of the worksite program into the organization’s structure, linkage to related programs, and worksite screening programs and related services [17].

The primary model of medical care insurance for working-age adults in the United States is employer-funded, whereby the employer makes group health insurance available to employees and their family members. This model provides a financial incentive for some employers to encourage healthy lifestyles for employees, since the costs of medical care for insured employees affect insurance premiums and the employer may pay half or more of their cost [5]. However, group health insurance coverage is less often provided by smaller employer organizations than larger ones [5].

Group health is a common vehicle for U.S. employers to offer WHP programs to their employees. In 2016, 50% of employers nationwide offering health insurance also offered WHP programs, with large employers (more than 200 employees) again being more likely to provide these programs than smaller employers (83% vs. 46%) [5]. Obstacles to smaller employers offering WHP programs include a muted ability to manage health care costs, as they are rarely self-insured (a scenario in which any savings from reduced medical costs are realized directly by the company). Smaller employers have limited discretionary financial resources and limited ability to negotiate volume-based discounts with health promotion vendors. They also rarely have the infrastructure of a central human resources department or the skills and knowledge to implement a WHP program. Last, research on the effectiveness of worksite health promotion is based on large employers, so the optimal approach for smaller employers is not well documented [18].

To support improvement in health of working adults, 19 states in the US (Alabama, Alaska, Arizona, Arkansas, Colorado, Delaware, Indiana, Massachusetts, Minnesota, Nevada, New Hampshire, North Carolina, Ohio, Oregon, Rhode Island, South Dakota, Texas, Vermont, and Wisconsin) have initiated efforts to date to encourage employers to undertake WHP programs. All but Alaska, South Dakota, and Vermont provide technical assistance to participating employers. Other than Massachusetts, only three states (Arkansas, Colorado, and Wisconsin) provide seed funding to employers for program activities, and only Maine and Oregon offer a tax credit to some or all participating employers. From the available documentation, it appears that few or none of these programs target the smallest employers or engage and guide employers to select specific activities which correspond to employees’ stated needs and/or interests.

The Commonwealth of Massachusetts (MA) has a history of supporting workplace wellness initiatives. In order to understand the needs of MA employers, three Worksite Wellness Benchmarking Surveys were conducted by the Department of Public Health (DPH) (2008, 2011, and 2014). The results of these surveys helped to inform programmatic aspects of the pilot program in 2008, as well as the current model described here. For example, in the 2014 survey, employers identified lack of available financial resources as a barrier to implementing worksite wellness programs [19]. The survey also revealed that MA employers were not evaluating health promotion programming. About 90% of respondents did not have a written strategic plan for worksite wellness; 81% did not set organizational objectives for health promotion; and many employers cited lack of knowledge about worksite health promotion as a barrier to implementing programs.

In 2008, Massachusetts launched “Mass in Motion,” a multi-sector, statewide obesity prevention initiative designed to help create conditions for healthy living in the places where people live, learn, work and play [20]. One component of Mass in Motion was a pilot worksite wellness capacity-building program, offered in 2008–2013, called “Working on Wellness” (WoW) (version 1.0). Employers received educational content through a delivery format of four full-day, in-person training programs over 12 months, along with individual technical assistance provided to each participating employer. This program reached over 60 MA employers with over 55,000 employees [21].

Experience from the pilot Mass in Motion program generated a number of lessons regarding elements of workplace wellness program design and employer recruitment efforts:

- Employers were interested in WHP programs but needed support from experts.
- The in-person training model was time-intensive and expensive to deliver.
- Recruitment techniques would need to reach significantly larger numbers of employers for meaningful public health impact.
- Financial resources, especially for small employers, are required to encourage participation.
- Employers want opportunities to share their experiences and lessons learned.
- Evaluation should be built in from the program’s inception.

Comprehensive health reform in Massachusetts was enacted in two phases. The second one (“Chapter 224”), in 2012, focused on improving the quality of medical care, reducing prevalence of preventable health conditions, and reducing health care costs. Among other provisions, Chapter 224 established a Prevention & Wellness Trust Fund (PWTF), investing $57 million into population-based clinical care coordination and health promotion efforts. The Trust supports community-based partnerships comprised of municipalities, healthcare systems, businesses, regional planning organizations, and schools. The partnerships are charged to provide research-based interventions to reduce rates of the most prevalent and preventable health conditions; increase the adoption of workplace-based health management programs; and address health disparities in the occurrence and management of chronic conditions.

Ten percent of the PWTF was designated for worksite health promotion programs, which led to the creation of a large-scalecapacity-building program building on the Mass in Motion employer pilot program and the 2014 Benchmarking survey. This program, “Working on Wellness” version 2.0, was designed to help businesses to implement comprehensive, evidence-based worksite health initiatives to create the conditions that enable and support employees to engage in healthy behaviors. While the program is not explicitly designed as a safety or hazard removal initiative, employers may also choose to address those aspects that intersect with health promotion under the program framework. WoW version 2.0 was offered to employers with customized coaching by program delivery staff and multiple reporting requirements to permit program evaluation. Other than the interactive activities (below) and data submission requirements, most of the program infrastructure remains publicly available as WoW version 3.0, a self-directed, on-line curriculum and catalog of information resources (https://mawow.org/). (The WoW program is also ongoing in that numerous data analyses of version 2.0 are initiated but not complete.)

Chapter 224 also established a Small Business Wellness Tax Credit program, offering MA businesses with 200 or fewer employees a state tax credit of up to 25% of the cost of implementing a qualified wellness program ($10,000 maximum). To qualify for the tax credit and receive certification, employers must meet certain eligibility criteria (e.g., offer health benefits to all employees, provide legally required workers’ compensation insurance) and agree to conform with program requirements (e.g., implement evidence-based interventions) [22]. The eligibility criteria for WoW were intentionally aligned with the eligibility criteria for the Small Business Wellness Tax Credit program, to maximize the opportunities for small employers in the state.

### Program aims and objectives

The goals of the capacity-building model for the WoW program are to:

- Increase the number of workplaces that have identified an on-site worksite wellness sponsor, champion, and team to formulate, implement and evaluate a Worksite Wellness Action Plan.
- Increase the number of workplaces that have a documented strategic plan for their WHP program.
- Increase the number of employers implementing policies and environmental changes that support healthy behaviors.
- Increase the number of new collaborations between employers and community organizations.
- Increase the proportion of employees within each participating workplace who:
  - Have knowledge and skills for healthy behavior choices
  - Participate in worksite education and behavior change programs
  - Participate in designing solutions in the workplace to address identified health concerns

This paper describes the WoW 2.0 program and the plan for its evaluation. Key evaluation questions are the success of WoW 2.0 in recruiting small and mid-size employers; the extent to which it achieves the above goals; and understanding the needs of smaller employers and industries not traditionally implementing employee wellness programs.

## Methods

### Program overview

Working on Wellness provides training, technical assistance and other support services to participating Massachusetts employers. The program seeks to build the skills, knowledge and capacity of employers and to promote employee engagement and retention. The program also emphasizes community linkages and partnerships, to encourage collaboration between workplaces and community organizations to help employers enhance their wellness programming. During WoW 2.0, program staff worked with participating employers for approximately 10 to 12 months to support them in developing an infrastructure to address the needs of the organization and its employees. Enrollment was controlled and data collection activities were included throughout the program, with real-time feedback, to facilitate customization to local circumstances and evaluation both in real-time and post-intervention.

### Program framework

**Fig. 1:**
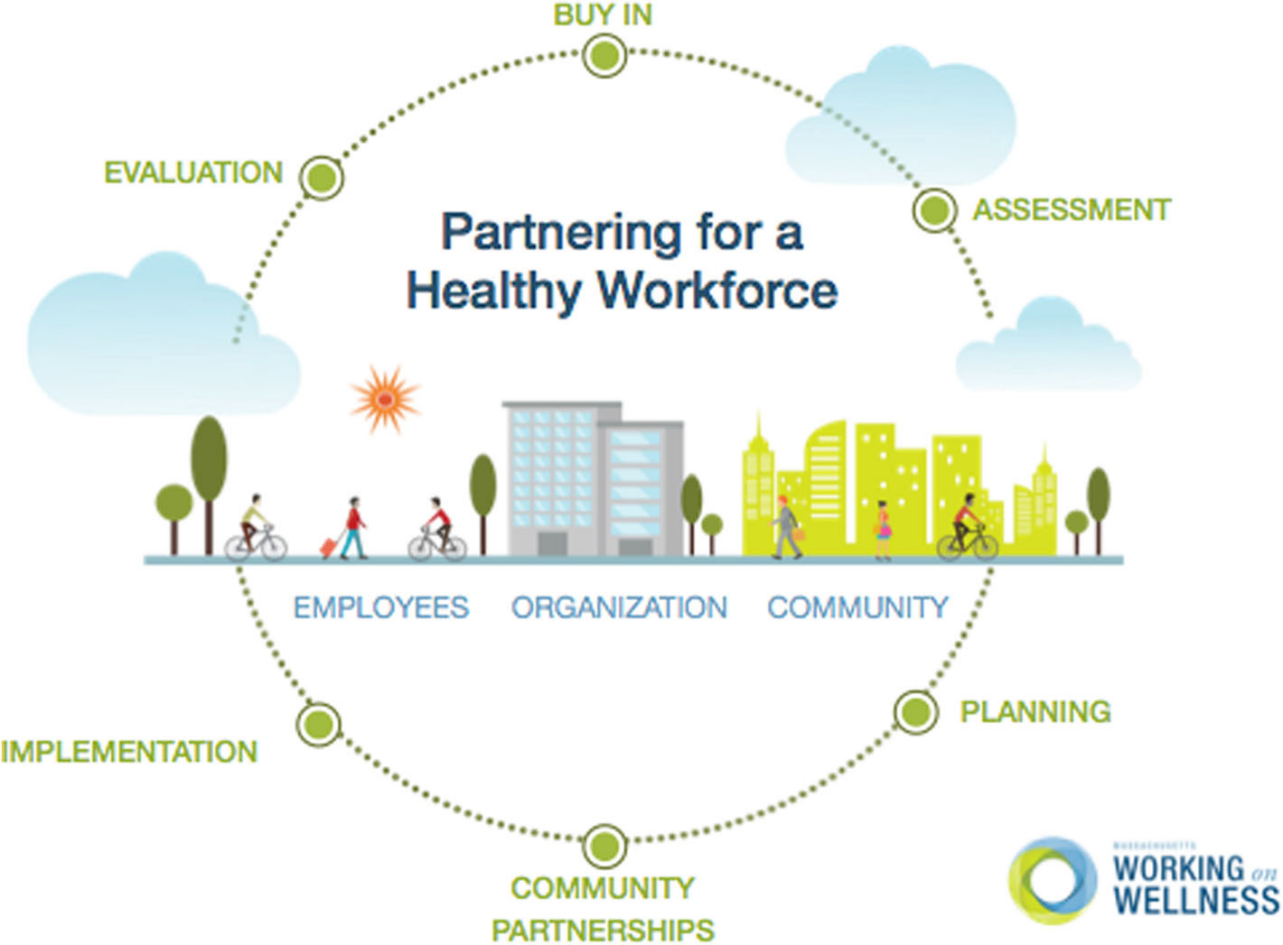
Program Development Cycle

Based on documented good practices for worksite wellness [17], the program curriculum follows a six-step program development cycle (Fig. 1).

The content areas of the Development Cycle are:

- Buy-In – Participants learn how to gain support from all levels of the organization, including senior leaders, managers and employees.
- Assessment – Employer organizations identify where to focus their wellness initiative. Standardized tools were provided to collect data on the organizational environment and employee health needs and interests.
- Planning - Participants use data gathered during the Assessment phase to create a strategic plan that meets the organization’s needs.
- Community Partnerships - Organizations are provided the training and tools to identify existing or new community partnerships and foster those relationships.
- Implementation - Organizations execute their plan by facilitating programs, changing workplace policies, and changing their physical environment to support healthier behaviors.
- Evaluation - Organizations look at their planned goals and objectives to determine if they were met and identified areas for improvement.

### Employer eligibility, recruitment, and enrollment

For WoW 2.0, employer organizations were eligible to apply if they met all of the following criteria:

- Is a for-profit or non-profit corporation, or a public sector entity, located in Massachusetts.
- Offers health insurance benefits to employees.
- Has over 50% of employees working in Massachusetts.
- Is in compliance with all legal obligations of employers including, but not limited to, those enumerated in Massachusetts General Laws (e.g., carry workers’ compensation insurance for employees).
- Does not have a comprehensive wellness program, i.e., one available to all employees which includes an explicit program plan, assessment of employee needs and interests, awareness and education programs, behavior change programs, and workplace policies.
- Has not already received a seal of approval for the wellness program from the Massachusetts Department of Public Health, under the Small Business Wellness Tax Credit Incentive Program.

Numerous recruitment strategies were used to publicize the program to employers across Massachusetts. A network of 200 outreach partners in a variety of industries was developed and engaged to promote WoW via their mailing lists, reaching 56,000 people. Awareness of the program was also built via highway billboard advertising, in-person meetings with chambers of commerce, paid advertisements on Facebook and LinkedIn and use of Twitter. Seventy free informational webinars about the program were presented, attracting 760 attendees. Program staff held 33 in-person meetings with employers, which were attended by 850 people. The MA DPH set a maximum target of 400 employer organizations, based on the available program resources.

While all Massachusetts employers were eligible to apply, specific effort was made to recruit employers with 200 or fewer employees; those in sectors employing a large population of low-wage workers [23]; and those located in communities that have received prior public health funding investment from Mass in Motion or the PWTF.

Organizations were enrolled in WoW 2.0 in four staggered cohorts over a one-year period. Employers interested in participating in the program were required to complete a short on-line application and attend an informational webinar. Eligible employers were notified of their acceptance into the program within 2 weeks of the application submission.

Within 1 month of being accepted, each organization was required to submit a Memorandum of Understanding signed by a senior executive, acknowledging willingness to complete the program requirements in order to be eligible for seed funding. (At this point, small for-profit employers were also reminded of their eligibility for the Massachusetts Small Business Wellness Tax Credit.)

In Cohort 1, employer organizations were asked to establish three programmatic goals and to undertake three to six activities for each of those goals (9–18 activities in total). Through the experience of providing technical guidance to employer Wellness Champions, it became apparent that program planning to address three goals meaningfully within the one-year program was too ambitious for new trainees. Therefore, in Cohorts 2–4, the program was revised to reduce the number of required goals from three to one. Employers were directed to select the program goal(s) based on a defined assessment process which combined data about existing organizational supports for health behaviors and employees’ self-reported health and program interests.

### Program resources for employers

WoW provides a self-paced learning curriculum, technical assistance, planning and assessment tools, and seed funding (see below). Some of these resources and tools remain accessible to employers in WoW version 3.0. Selected tools and resources are also available to the general public (including non-eligible employers), such as an on-line resource database and the expert webinar series.

#### Training curriculum

The Working on Wellness curriculum was developed using the six-step Program Development Cycle and delivered via an on-line learning management system. Within 2 weeks of an employer’s notification of acceptance into the program, access was provided to the on-line learning portal. A detailed schedule of the learning curriculum and key benchmarks was provided to all participants. Each participating organization was asked to commit two individuals’ time to view the on-line curriculum and to use the tools provided on the WoW website to develop and implement the wellness program plan.

Throughout the program, organizations were reminded that their interventions should include organization-level changes in policy (e.g., catering standards) and physical environment (e.g., stairwell beautification and lighting), as well as those targeting individual behavior change, such as raising awareness and education.

#### Technical assistance

In each cohort of WoW 2.0, the employers were grouped for technical assistance based on size or similar industries. Bi-weekly technical assistance conference calls with workplace wellness subject matter experts reinforced the learning process and provided an additional level of support. During each technical assistance call, several polling questions were used to gauge participants’ experience with the program and to inform on-going quality improvement efforts.

#### Assessment tools

Employers are provided with standardized self-assessment and data collection tools. The Environmental Scan gathered information on the workplace physical attributes, and health-related policies and programs. The Needs and Interest survey collected information from individual employees on current health status and behaviors, program areas of interests, and readiness for changes. In WoW 2.0, summary data from both of these instruments were reported back to each employer for their own use in developing a customized action plan. Each employer organization was guided to develop strategic goals, an action plan, and corresponding interventions to meet the needs and interests of its employees.

#### On-line support resources

In addition to the curriculum and technical assistance, a searchable database of worksite wellness resources was created with over 375 free, publicly available resources from government and non-profit sources.

#### Webinar series

An expert webinar series offers access to subject matter experts on a wide variety of topics, such as ergonomics, indoor air quality, diabetes prevention, stress, physical activity, mobile health clinics, and healthy eating.

#### Seed funding

Each employer enrolled in WoW 2.0 was eligible to receive up to $10,000 in seed funding, a percentage of which had to be matched by the employer. Private businesses were required to match 100% of the amount of their seed funding; up to 100% of that match could be in-kind resources. Government entities and nonprofits must match 50% of their seed funding, again with up to 100% as in-kind.

Seed funding was dispersed in 3 separate payments of $2000, $7000, and $1000, contingent on achieving a series of defined benchmarks: appoint a wellness champion and wellness committee members; establish a program budget; conduct an environmental audit; conduct the baseline and follow-up employee Needs and Interests (N&I) surveys; complete on-lineself-paced curriculum; submit a Worksite Wellness Action Plan; attend expert webinars; and submit a Worksite Wellness Evaluation Report (WWER). For the third payment, employers could choose to receive half of the full payment for completing either of the two last benchmarks, the follow-up N&I survey or the WWER.

#### Employer sharing forum

This annual event provided an opportunity for program participants to come together in person and learn from each other’s experience. This was held after all four cohorts of WoW 2.0 participants had been recruited. Selected early participants were invited to present on their experiences and programs.

### Evaluation

A variety of data collection points were established, including recruitment and benchmark milestone achievements (Table 1). The instruments were provided to employers to collect data for use in coaching of participant organizations, assessing participation levels, and evaluating effectiveness of the interventions implemented, both in real time and afterwards.

**Table 1:** Data collection instruments for “Working on Wellness” program implementation and evaluation

Instrument	Source of information	Time of administration	Key measures
Program application	Employer representative	Baseline	Economic sector; workforce size, estimated turnover and proportion low-wage employees; employer readiness to participate
On-boarding survey	Employer representative	Baseline	Workforce demographics; conditions of work (e.g., shift schedules); current wellness activities offered
Non-participant survey	Employer representative	Post enrollment deadline (Cohort 1 only)	Top reasons for not participating; opinions of the program; recommendations for future programs
Exit interviews	Employer representative	After enrolled employers withdrew	Primary reasons for withdrawal; barriers to participation and implementation
Employee Needs and Interests survey	Employee self-administration	Baseline; End of program	Health/disease conditions; health behaviors; overall health risk; wellness topics and activities of interest
Environmental Scan	Employer representative	Baseline	Current employee health, safety, and well-being policies and programs
Worksite Wellness Action Plan	Employer representative	During program (after key curriculum milestones)	Program planning: budget, goals and objectives, intended interventions, community partners, and resources
Worksite Wellness Evaluation Report	Employer representative	Post-intervention implementation	Types of interventions implemented (programs and policies); spending
Process evaluation group interviews with WoW personnel	Program delivery staff members	After each cohort’s program	Programmatic successes, challenges, and recommendations for change: planning, recruitment, survey administration, project management, etc.
Key informant interviews	Wellness Champions at participating organizations	Post-intervention and at least 1 year later (Cohorts 1 and 2 only)	Types of interventions implemented (programs and policies); spending. Usefulness, value, involvement, and satisfaction levels, recommendations for improvements, challenges, sustainability

Both baseline and follow-up measures were collected at the employer and employee levels. A logic model was developed to guide the program design and evaluation approach (Additional file 1).

Evaluation of the WoW program includes both real-time and pre-post assessments. The short-term results informed some program revisions between cohorts, to improve enrollment and facilitate improved compliance with program requirements. For example, interviews with program delivery staff identified several challenges, resulting in adaptations such as more centralized tracking of employer recruitment and applications; revised materials to clarify expectations of employers; slightly longer timeline to give prospective applicants time to assess the program; more customized recruitment materials to target under-represented industries and those with low-wage workers; expanded contact networks; and stronger marketing plan, including success stories from Cohort 1 participants. The reduction in number of required health goals from three to one (see above) is another example of a real-time revision based on early feedback. All of these changes were announced publicly through the online WoW program materials.

Longer-term evaluation activities will utilize data collected from these instruments to characterize the following:

- Effectiveness of recruiting and retention efforts, including with regard to representativeness of participating employers by size and sector for Massachusetts;
- Willingness of MA employers, especially small businesses, to implement comprehensive worksite wellness programs;
- The extent to which employers utilized the seed funding incentive to commit staff time and resources to the program;
- The extent to which small employers utilized the tax credit to finance program implementation;
- Perceived usefulness of technical assistance provided to groups of employers;
- Short-term improvements in employee health behaviors (workplace-level change);
- The extent of new linkages created between employers and local health-related community resources.

These questions will be addressed with a mix of quantitative and qualitative data, through descriptive analyses at different points in time and pre-post comparisons. Employer plans and actions, by definition, will be among organizations that did not have comprehensive programs at baseline.

## Discussion

This innovative program introduces organizational level capacity-building as a way of achieving worksite health promotion. Unlike other models, this model is focused on generating changes at the organizational level, not only at the individual employee level. Program design and the curriculum delivered to employers were based on well-grounded public health prevention practice models relevant in the fields of chronic disease prevention (e.g. the CDC Health Impact Pyramid, the World Health Organization [13]) and occupational safety and health (e.g. the NIOSH hierarchy of controls). These models emphasize population-level policies and system changes in which the interventions can benefit most of the population and set the conditions for successful behavior change on the part of individuals.

To meet the challenge of sustaining these programs beyond the intervention period, WoW 2.0 participants were required to establish an internal infrastructure (Champion and Committee) to plan and manage the program, and to form community partnerships. Particularly in light of the fact that program recruitment targeted small businesses, these community connections were intended to provide linkages to local resources and services that could be incorporated into the program, as a partial solution to the lack of internal staffing specialists and other resources.

A strength of this approach is the program’s ability to reach a large number of employers through the use of on-line materials and group technical assistance. Further, the seed funding addressed an unmet need for smaller employers who might not be able to fund such a program themselves.

The evaluation protocol includes numerous points of data collection that were designed to be of use to the participating employers and program providers in real time (as well as to evaluators). For example, each employer was provided with an individual summary report of employee self-reported health needs and interests data, which was to be used to customize program prioritization and planning. Employers were taught how to use evaluation instruments and data to track their program process and outcomes during the program period. Some of the instruments (e.g., Worksite Wellness Evaluation Report) remain available to employers after the program period ended for their own use in ongoing program evaluation and monitoring.

The opportunity for real-time corrections was another strength of the program. The enrollment of staggered cohorts allowed for ongoing program review and quality improvements; initial review of experience with the early cohorts could inform program design features to enhance enrollment and participation, as described above.

One notable limitation of program design is the short time frame for direct involvement with each cohort of employers, originally intended to be 10 months. For these employers with no prior WHP experience, it was time-consuming to coach them through the stages of start-up, including initial assessment and evidence-informed program planning. The third and fourth cohorts had engagement periods extended up to 15 months, but this was still insufficient to provide satisfactory coaching through the later phases of self-evaluation, strategizing about how to build on and revise the initial plan, and planning for program sustainability. A related issue was the short time frame available at start-up (due to contractual procedures) for developing and launching the program. This might also have limited the ability to recruit and enroll organizations, despite expanded efforts for the later two cohorts.

A caveat regarding the evaluation plan is that the N&I surveys will primarily be used for metrics of workplace-level change. This is consistent with the WoW design and purpose, which focuses on the workplace as the locus of change. It also reflects the nature of the collected data, as the surveys were completed anonymously by employees on either work time or personal time. Baseline response rates were low to moderate, and some organizations have moderately high annual turnover rates; both of these would hamper analysis of within-person changes. Further, while respondents were guided to create a unique personal identifier in the baseline survey, failure to reproduce or enter it correctly in the follow-up would also interfere with survey pairing and individual-level analyses.

The WoW program emphasizes environmental and institutional changes at the worksite, with the expectation that system-level changes might lead to more sustainable initiatives and long-lasting behavioral changes by individuals. However, it does not directly address the influence of working conditions, which can affect both health behaviors [9–12] and worker program participation [24, 25]. Unhealthy work environments are more common for some population groups; in particular, low-wage workers are more frequently exposed to environmental toxins, safety hazards, and psychosocial stressors in the workplace [8, 26]. We will attempt to examine whether the prevalence of such exposures exerted any influence on individual participation, readiness for change, or health behaviors.

Workplace HP programs are voluntary on the part of employers in the U.S. Organizations may be more willing to invest resources in full-time and higher-paid workers whom they wish to retain and less likely to do so for the proportion of the workforce that is low-wage or contingent. However, it would also be of public health value to develop strategies to reach worksites with low-wage and part-time workers, who may not have group health insurance or paid sick leave, let alone health-promoting environments, as well as those whose work or worksite itself is hazardous. The current WoW model has not yet been extended specifically to address those underlying issues.

Another important question is whether this model is best suited for employers with a central physical location. We have not yet considered whether it would need to be adapted for sectors with a distributed workforce, such as home health aides, construction workers, or commercial drivers.

It is hoped that, over the longer term, WoW might stimulate more employers to offer comprehensive wellness programs to their employees and eventually help to reduce the burden of chronic disease and lower health care expenditures in MA. This model may provide lessons for MA and other state departments of health in how to achieve sustainable and effective collaborations between government public health agencies, employers, and local community service providers to improve workforce health.

## Additional file

Additional file 1: Logic Model for the “Working on Wellness” program (DOCX 30 kb)

## Abbreviations

DPH: Department of Public Health
MA: Massachusetts
N&I: Needs and Interests
PWTF: Prevention and Wellness Trust Fund
US: United States
WHP: Worksite health promotion
WoW: Working on Wellness
