# Supplementary material for: “Working on Wellness:” protocol for a worksite health promotion capacity-building program for employers
Source: BMC Public Health. 2019 Jan 25;19:111. doi: 10.1186/s12889-019-6405-1 (PMC6347764; doi:10.1186/s12889-019-6405-1)
Supplement: Supplementary file 1 — Logic Model for the “Working on Wellness” program (DOCX 30 kb) [file 12889_2019_6405_MOESM1_ESM.docx]

**MA Department of Public Health -- Prevention and Wellness Trust Fund -- Worksite Wellness Project**

**VISION STATEMENT:**

**smaller workplaces HAVE the requisite KNOWLEDGE, skills, strategies, AND/or resources to adopt sustainable policies and Practices that PROMOTE AND PROTECT THE HEALTH OF EMPLOYEES**

| **INPUTS** | ***STRATEGIES* + OUTPUTS** | **OUTCOMES** | | | Decreased health care costs and workers’ compensation claims.  Decreased premature morbidity and mortality.  Decreased prevalence of hypertension and other chronic diseases. |
| --- | --- | --- | --- | --- | --- |
| ***Personnel****:*   - HRiA/AdvancingWellness staff - MDPH/PWTF staff - Subcontractors (e.g. 4WebInc.) - Other partners (e.g. Chambers, Unions) - Businesses   ___________________________________________  ***Project Management Systems****:*   - Web-Based Workplace Portal - Outreach/Recruitment/ Enrollment - Grants Administration - Learning Management System - Training/TA - Benchmark/Milestone Reporting - Employer MOUs   ***Existing Program Resources****:*   - Employer assessments - Educational material/ Resources - Technical guidance and literature on evidence-based practices and promising approaches   ___________________________________________  ***Environmental Scan***:   - Tax credit history - Employer Needs/Interests survey - National Healthy Worksite Initiative - DPH Working on Wellness program - Prevention Wellness Trust Fund communities   ___________________________________________  ***Financial Resources***:   - Seed funding - MA Wellness Tax Credit for Small Businesses - Prevention Wellness Trust Fund - Employer contribution   ________________________________________  ***Evaluation***:   - Evaluators (e.g. CPH NEW, UMASS Medical, UMASS Lowell) | ***Develop and implement outreach, recruitment, and marketing strategy:***   - Outreach & Recruitment Plan | **Short-Term (1-3 yrs)** | **Intermediate (3-5 yrs)** | **Long-Term (5-10 yrs)** |  |
|  |  | ***Increased # of workplaces with an on-site worksite wellness sponsor/champion/team.***  ***Number/type of organizational levels represented in the process of developing a Worksite Wellness Action Plan (WWAP)***  ***_________________________________***  ***Increased # of workplaces with a WWAP***   - # new policies - # new practices - # environmental strategies   ***_________________________________***  ***Increased # of workplaces applying for small business tax credit***  ***Increased # of employees:***   - Increasing knowledge about healthier living - Participating in worksite education and behavior change programs - Changes in social norms   _______________________________  ***Increased # of new business - community collaborations***  _______________________________  ***# of businesses with non-traditional populations participating in this initiative that meet the following targeted conditions:***   - Fewer than 200 employees - Located in MiM, PWTF, and 1422 communities - Lower wage workforce - Identified target industries | ***Increased number of sustainable workplace wellness programs in MA small businesses.***    [The remainder of the intermediate outcomes are outside the scope and term of the planned evaluations.]  ***Increase in positive employee behavior change:***   - Healthy eating/Active living - Smoking cessation - Use of primary care - Stress management - Hypertension-specific management   _______________________________  ***Decreased % of employee exposures to unhealthy or unsafe work conditions:***   - Secondhand smoke - Chemicals - Unhealthy foods - Ergonomic hazards   ***Employment Outcomes:***   - reduced sick days - decrease in employee turnover - Improved productivity | [Long-term outcomes are outside the scope and term of the planned evaluations.]  ***Improved employee health status, especially related to hypertension and related conditions.***  ***Increased equity in workplace health conditions***  ***Reduced disparities among employees:***   - Reduced injuries to low income and minority workers - Reduced disparity in behavior change, exposures, satisfaction (including autonomy), and productivity |  |
|  | ***Develop and implement grants administration process:***   - Information Sessions/ webinars conducted - Worksite applications reviewed/accepted - Employer MOUs in place - Worksite benchmarks/ Milestones reported - Funds distributed - Worksite Action Plans |  |  |  |  |
|  | ***Provide training, tools, and resources to targeted worksites through web-based portal/annual best practices forum***:   - 350 worksites enrolled; (75 worksites in cohort 1; 125 in cohort 2; 150 in cohort 3) - 350 worksite environmental audit conducted - Employee-level needs and interest data collection - Training Curriculum/Calendar - Selected worksites receive: - # webinars; % worksites participating in webinars; % participant satisfaction - # self-paced modules; % worksites successfully completing modules; % participant satisfaction - % of worksites participating in online learning community - # resources/tools accessed - % worksites accessing web-based resources - % worksites providing/ participating in mentoring opportunities - % worksites sharing best practices and resources - # worksites participating in annual best practices forum |  |  |  |  |
|  | ***Provide TA on evidence-based policies/practices:***   - # hours of clustered/group TA provided based on worksite size/sector/geographic location/need/capacity, etc. - # Worksite Action Plans reviewed; % plans that meet worksite wellness guidelines - # Correction plans developed - # hours of group remediation TA provided |  |  |  |  |
|  | ***Disseminate timely and accurate information/resources to targeted worksites:***   - X blogs - X fact sheets/case studies developed on key topics - X tools developed/shared - X newsletters - X brochures/pamphlets distributed |  |  |  |  |
|  | ***Provide training and tools for assessment and evaluation at individual business level:***   - Needs/Interest Survey - Environmental Scan - Evaluation Plan   ***_____________________________________________***  ***Foster linkages in community between businesses and local resources.*** |  |  |  |  |
